# Optimizing the Evaluation of Letters of Recommendation for Obstetrics and Gynecology Residency Applicants: Development and Validation of a ChatGPT-Powered Tool

**DOI:** 10.1097/og9.0000000000000049

**Published:** 2024-12-05

**Authors:** Pedro Morales-Ramirez, Erick Gutierrez, Brettany DeMier, Preslie White-Hammonds, Henry Mishek, Arhita Dasgupta

**Affiliations:** Department of Obstetrics and Gynecology, University of Missouri-Kansas City, Kansas City, Missouri; and American University, Managua, Nicaragua.

## Abstract

**OBJECTIVE:**

To develop and validate a ChatGPT-powered tool for assessing letters of recommendation in obstetrics and gynecology residency applicants.

**METHODS:**

This study was conducted in two phases at a university-based obstetrics and gynecology residency program. During the development phase, 30 letters of recommendation were used to create ChatGPT prompts based on a validated scoring rubric that assesses phrases, letter features, and applicant abilities. ChatGPT scores were compared with benchmarks using intraclass correlation coefficients (ICCs) to evaluate reliability, and ChatGPT processing times were measured against those of human reviewers. In the second phase (validation), 88 letters of recommendation from residents with and without academic deficiencies (n=30) were analyzed using the ChatGPT prompts developed and refined in the first stage. Residents with academic deficiencies (case group) were matched 1:1 with a control group of residents without noted deficiencies by medical school, gender, and training period. The letter of recommendation scores between the case and control groups then were compared using the Mann-Whitney U test.

**RESULTS:**

ChatGPT significantly reduced review time per letter, from 7.2 minutes to 45 seconds. Inter-rater reliability between ChatGPT and benchmark scores indicated strong agreement for specific phrases (ICC 0.82, 95% CI, 0.75–0.88), moderate agreement for letter features (ICC 0.75, 95% CI, 0.68–0.82), and fair agreement for applicant abilities (ICC 0.70, 95% CI, 0.62–0.78). After refinement, ICCs improved to 0.92 (95% CI, 0.88–0.95), 0.81 (95% CI, 0.74–0.87), and 0.77 (95% CI, 0.69–0.84). Mean letter of recommendation scores for specific phrases differed significantly between residents with deficiencies (4.49; 95% CI, 3.29–5.68) and residents in the control group (6.00; 95% CI, 5.00–7.00) (*P*=.017). Subgroup analysis showed an even greater gap between residents with multiple deficiencies (2.15; 95% CI, 1.42–3.38) and residents in the control group (6.00; 95% CI, 5.00–7.00; *P*<.001), suggesting a higher predictive value for at-risk residents.

**CONCLUSION:**

ChatGPT can enhance the residency selection process by providing reliable letter of recommendation analysis significantly faster than human reviewers and effectively identifying key phrases in letters of recommendation that could predict future academic deficiencies.

## Abstract 2

A ChatGPT-powered tool can enhance efficiency and consistency in evaluating obstetrics and gynecology residency applicant letters of recommendation.

Letters of recommendation are among the top three factors residency program directors consider when selecting applicants for interviews.^1^ They offer valuable insights into an applicant's noncognitive skills, professionalism, and overall suitability for a residency program. With the transition of USMLE step 1 to a pass–fail system, the importance of letters of recommendation and other qualitative data are expected to grow as part of a holistic review process.^2–5^ However, despite their importance, letter of recommendation interpretation suffers from low inter-rater reliability,^6,7^ largely due to the lack of uniform assessment guidelines. Although the introduction of standardized letters of evaluation has mitigated some of the limitations of traditional letters of recommendation, they still exhibit poor overall reliability across specialties.^8–10^ Given these challenges, it is important to conduct research aimed at improving letter of recommendation reliability as part of the shift toward holistic reviews.

The emergence of large language models (LLMs), such as ChatGPT, offers a promising solution to the challenges of evaluating letters of recommendation by providing more consistent and less biased assessments.^11,12^ Although traditional natural language processing (NLP) and machine learning techniques have been applied to letter of recommendation analysis and other aspects of residency applications, none of the published studies have used LLMs such as ChatGPT.^13–20^ Traditional NLP methods can identify patterns and keywords in text but lack the ability to fully understand context and nuance. Additionally, they require specialized software and trained personnel, limiting their accessibility. In contrast, LLMs such as ChatGPT can capture context, infer meaning, and handle complex language structures that traditional NLP models struggle with. Most LLMs are freely available and require no prior coding knowledge, making them not only accessible but also highly scalable. Because program directors often lack the time to thoroughly evaluate narrative content in applications, tools such as ChatGPT can assist in streamlining and enhancing holistic review processes. This highlights the need to explore the capabilities of these accessible LLMs, because they have the potential to empower residency programs to leverage this technology and improve the efficiency and effectiveness of application reviews.

Our study addresses the gap in research on LLMs in resident selection by developing and validating a process that leverages ChatGPT's advanced capabilities alongside a validated letter of recommendation scoring rubric.^20^ To validate our model, we used a case–control design with resident performance metrics defined by an established Clinical Competency Committee in a real-life clinical environment, contrasting with the hypothetical academic outcomes used in earlier letter of recommendation research.^21–23^ Our goal is to enhance the residency selection process by providing an artificial intelligence (AI)–powered tool that allows a consistent and reproducible process to interpret these letters. By reducing evaluation time, improving consistency, and decreasing bias, LLMs have the potential to enhance residency selection, making it more efficient and equitable.

## METHODS

This study was conducted at a university-based obstetrics and gynecology residency program with eight residents per class. It was carried out in two phases—development and validation—from January to July 2024, with the second phase involving a retrospective analysis of resident data from July 2013 to July 2023. The project was exempted by the University of Missouri-Kansas City IRB, because it involved research in established educational settings and did not require identifiable participant information (UMKC IRB#2101704).

ChatGPT prompts for letter of recommendation analysis were developed by integrating a published framework for qualitative research^11^ with a validated letter of recommendation scoring rubric.^20^ The framework involves designing prompts for task background, task descriptions, and output format, followed by iteration, refinement, and human oversight of the initial prompts. The integrated rubric, developed by a group of medical educators based on national surveys and previous research, consists of three sections: “commonly used phrases” (21 phrases, scored −2 to 2, with a minimum score of −10 and a maximum score of 14), “letter features” (four features, rated 1 to 5, with a minimum score of 4 and a maximum score of 20), and “applicant abilities” (14 abilities, rated 1 to 3, with a minimum score of 14 and a maximum score of 42). Inter-rater reliability was measured using intraclass correlation coefficients (ICCs), and the rubric showed strong reliability for section 1 (specific phrases) (ICC 0.82) moderate for section 2 (letter features) (ICC 0.60), and low for section 3 (applicant abilities) (ICC 0.28) (Appendix 1, available online at http://links.lww.com/AOG/D926). For our study, we considered an ICC lower 0.50 to indicate poor reliability, 0.50–0.75 moderate reliability, and greater than 0.75 strong reliability.^24^

Initial prompts were created for background, tasks, and output, and these were saved under a customized “GTP” we called “LOR analyzer” (available at https://chatgpt.com/g/g-sV2U3Vf3s-lor-analyzer). These initial prompts served as the baseline for subsequent prompts in all three sections of the rubric (Table 1). For the iteration, refinement, and human oversight part of the framework, prompts for each section of the rubric were developed individually (Appendix 2, available online at http://links.lww.com/AOG/D926). Benchmark scores were developed for 30 randomly selected letters of recommendation, chosen to represent the typical variety and complexity of such letters. Two investigators participated in a structured training session led by the principal investigator that included comprehensive instructions on rubric application, practice scoring using case studies, and calibration sessions to ensure consistency in their evaluations. A benchmark score was automatically assigned if the principal investigator and at least one reviewer independently provided the same score for a letter. In cases in which this agreement did not occur, a benchmark score was determined through consensus. The rationale for all benchmark scores was documented to ensure this information could be used to train ChatGPT effectively. The goal of this stage was to create a “gold standard” for training and evaluating ChatGPT, based on an already validated human instrument, rather than to assess ICC among the three reviewers. The same 30 letters of recommendation were then analyzed using the initial ChatGPT prompts (Appendix 3, available online at http://links.lww.com/AOG/D926). For any instances where ChatGPT's ICC fell below 0.75 when compared with benchmarks, ChatGPT's metacognitive capabilities were engaged to provide an explanation for its assigned score (Appendix 4, available online at http://links.lww.com/AOG/D926). The human-generated benchmark scores, along with detailed explanations, were provided to ChatGPT, which was tasked with identifying the source of the discrepancy and refining its prompts to improve alignment with the human scores. During this stage of refinement, ChatGPT suggested incorporating scoring examples, which were then integrated into the revised prompts. Through this iterative process—where human benchmark scores and rationale informed ChatGPT's adjustments—both the algorithm and prompts were progressively refined.

**Table 1.** Final Prompts Created During the Development Phase

Step	ChatGPT Prompt	Comments
Task background	“You are an experienced qualitative researcher, and your expertise includes content analysis of unstructured data in residency applications like letters of recommendation.”	These 3 prompts were built into the ChatGPT we named “LOR Analyzer”
Task description	“Your task is to systematically analyze sentence by sentence, the content of each letter of recommendation provided to you. Your main goal is to ensure that the analysis is consistent and reliable. If the same letter would be provided in a new chat, the output should be the same. You will analysis letters of recommendation using a provided validated scoring rubric. You will be given specific instructions for each section of the rubric in a different chat”	
Output format	“Write the output in two sections. For the first section, write each sentence of the letters, explain if it meets the criteria from the LOR rubric and explain why. For the second section, assigned the predefined scores for each sentence, add calculate a final score as specified by the instructions of that section of the rubric”	
Section 1: specific phrases	“Task #1: Analyze the letter content sentence by sentence and identify the following phrases: Tier 1 Phrases: Phrases that indicate the highest level of praise and outstanding performance (Examples: “Exceptional student,” “Asset,” “Outstanding student,” “Exceeded expectations,” “Highest recommendation,” “At the level of an intern,” “At the level of a resident,” “One of the best,” “Will try to recruit,” “Would like to stay,” “Would rate in the top 10”), Tier 2 Phrases: Phrases that indicate strong recommendation and high performance (Examples: “Excellent student,” “Highly recommend,” “Recommend without reservation,” “Would rate in the top quartile,” “Function at the level of a 4th year student”), Tier 3 Phrases: Phrases that indicate average performance (Examples: “Expected level,” “Adequate,” “Satisfactory,” “Acceptable,” “Appropriate,” “Improved”), Tier 4 Phrases: Phrases that indicate below-average performance or struggles (Examples: “Struggled,” “Had difficulties,” “Failed”) Each identified phrase will be counted only once per sentence to maintain accuracy. After identifying the phrases, cross-check them against their respective their categories to confirm accuracy. Task #2: Calculate the final score by assigning 2 points for each Tier 1 phrase, 1 point for each Tier 2 phrase, −1 point for each Tier 3 phrase, and −2 points for each Tier 4 phrase.	These prompts were added individually for each section, and examples from the benchmark scores were added to improve accuracy.
Section 2: letter features	“Task#1: Analyze each letter of recommendation. On a scale from 1 (no mention of the feature) to 5 (very detailed description), rate how well the letter writer described each of the following features: Feature #1: Description of the depth of interaction with the applicant, Feature #2: Description of the applicant's specific abilities/qualities, Feature #3: Summative statement of the strength of recommendation, feature #4: Inclusion of personal details about the applicant.” Use the provided examples to calibrate your ratings. Task #2: Calculate an average for this section using the scores of each feature	
Section 3: applicant abilities	“Task#1: Rate how well the letter writer described any of the following applicant abilities/qualities on a scale from 1 (none mentioned) to 3 (specific examples provided): Ability #1: Work ethic, hardworking, dedicated, motivated, conscientious, Ability #2: Trustworthy, patient ownership, reliable, responsible, Ability #3: Team player, collaborative, good to work with, helpful, Ability #4: Professional, integrity, role model, respectful, Ability #5: Compassionate, kind, empathetic, caring, comforting, bedside manner, Ability #6: Maturity, mature, life experience, Ability #7: Resilient, resilience, flexible, persevered, Ability #8: Leadership, leader, poised, innovative, took initiative, Ability #9: Resourcefulness, resourceful, quick learner, self-directed, independent, Ability #10: Inquisitive, curious, asked good questions, engaged, Ability #11: Good communication skills, interacts well with others, engaging, sincere, good listener, Ability #12: Clinical reasoning skills, good judgment, good differentials, good knowledge, solid knowledge, bright, Ability #13: Efficient, notes in time, efficient seeing patients, Ability #14: Enthusiastic, energy, motivation, proactive, proactiveness, without prompt, quick learner. Task #2: Calculate an average for this section using the scores of each feature	
Itineration and refinement	“The following letter was analyzed by 2 independent raters and ChatGPT in a different conversation. The reviewers gave a score of [human score] and ChatGPT's score was [ChatGPT score]. Explain the reasons for the discrepancies, and tell me how I should write the prompts next time to improve consistency: [letter text].”	

LOR, letter of recommendation.

The principal investigator and residency program director identified study participants by reviewing Clinical Competency Committee minutes for the study period. The program director then extracted letters of recommendation from their Electronic Residency Application Service files, removed identifiable information using Adobe Acrobat Pro, assigned random codes, and compiled the letters into a Word document. Chair's letters were excluded from the analysis, because 10% of applicants lacked one, and the remaining Chair's letters were mostly summaries without first-hand knowledge of the applicant. We also excluded Standard Letters of Evaluation, because they were not part of the application until the 2021–2022 cycle.

Once study participants were identified, all letters for the participants were analyzed using the revised prompts. The analysis was conducted using two separate ChatGPT accounts to cross-validate the results, limiting each chat to 10 letters to stay within ChatGPT's “context window” for effective analysis. We recorded average review times per letter for both human reviewers and ChatGPT, as well as ChatGPT's ICC values before and after refinement. ChatGPT 4.0 (March 2024) was used for all tasks.

A case–control design was used to apply the revised prompts generated in the development phase. The case group consisted of residents identified by the Clinical Competency Committee as having persistent academic deficiencies in one or more Accreditation Council for Graduate Medical Education core competencies, requiring interventions such as performance improvement plans or reportable actions from July 2013 to July 2023. Deficiencies had to be evident in at least two consecutive semiannual evaluations, thereby excluding isolated instances of underperformance that may have been caused by unforeseen circumstances (health, personal, or other issues). The control group consisted of residents without noted deficiencies, matched to residents with deficiencies in the case group based on medical school, gender, and training period within a 3-year window to minimize confounding factors such as variations in letter of recommendation writing practices among different medical schools, gender bias, and evaluation variations. The Clinical Competency Committee identifies academic deficiencies by reviewing data from formative and summative evaluations, peer and nursing staff assessments, and in-training examination scores.

Our primary objective was to compare the rubric scores calculated by ChatGPT for residents with academic deficiencies (case group) and those without (control group) across each section. A power analysis was performed using the first section of the rubric, which had the highest inter-rater reliability in the original study.^20^ The mean score for the lowest-performing group in that study was 2.3, which we hypothesized would match our case group. To prevent underpowering, we set a minimum mean score difference of 1 point between the case and control groups, calculating a sample size of 15 participants per group for 80% power. For the final analysis, all letters for each participant were scored and averaged to produce a single score per applicant. Due to the nonnormal distribution of letter scores, we applied the Mann-Whitney U test to compare scores between groups. The U statistic is reported alongside *P*-values (*P*<.05 is considered significant) to account for the rank-based group comparison. Additionally, we calculated Cohen's d to measure effect size, providing a more complete understanding of the statistical and practical significance of our results. All statistical analyses were performed in SPSS 27.

## RESULTS

We analyzed a total of 88 letters of recommendation from 30 participants, with 15 residents in each study group. Review times per letter were significantly reduced when using ChatGPT compared with human reviewers. For the first five letters, human reviewers took an average of 12.6 minutes per letter (95% CI, 11–14 minutes) to apply the three sections of the rubric. Although review times improved with experience, they plateaued after the 12th letter, resulting in a final average time of 7.2 minutes per letter (95% CI, 6–8 minutes). In contrast, ChatGPT took an average of 45 seconds per letter, including the time required to add prompts and input the letter content.

We compared the scores generated by ChatGPT using the initial prompts to the benchmark scores across the three sections of the rubric: specific phrases, letter features, and applicant abilities. For specific phrases, ChatGPT scored 5.40 (95% CI, 4.60–6.20) compared with the benchmark of 5.35 (95% CI, 4.60–6.10), with an ICC of 0.82. In letter features, ChatGPT had a mean score of 4.30 (95% CI, 4.10–4.50) compared with the benchmark score of 4.25 (95% CI, 4.05–4.45), with an ICC of 0.75, indicating moderate agreement. For applicant abilities, ChatGPT scored 2.58 (95% CI, 2.52–2.64) against the benchmark score of 2.58 (95% CI, 2.52–2.64), with an ICC of 0.70, showing a moderate, but acceptable, level of agreement. After iteration and refinement of the initial prompts, the ICC improved in all three sections, but the improvements in ICC were not statistically significant. The ICC for section 1 increased to 0.92: ChatGPT 5.50 (95% CI, 4.70–6.30) compared with benchmark 5.35 (95% CI, 4.60–6.10). For section 2, the ICC increased to 0.81: ChatGPT 4.40 (95% CI, 4.20–4.60) compared with the benchmark score of 4.25 (95% CI, 4.05–4.45). Section 3 improved to an ICC of 0.77, with ChatGPT scoring 2.60 (95% CI, 2.54–2.66) compared with the benchmark score of 2.58 (95% CI, 2.52–2.64) (Table 2).

**Table 2.** ChatGPT Intraclass Correlation Coefficients Before and After Itineration and Refinement

Scoring Rubric Section	Benchmark Scores (95% CI) (n=30)	ChatGPT Scores Before Itineration and Refinement (95% CI) (n=30)	ICC Before Iteration (95% CI)	ChatGPT Scores After Itineration and Refinement (95% CI) (n=30)	ICC After Iteration (95% CI)	*P*
1: Specific phrases	5.35 (4.60–6.10)	5.40 (4.60–6.20)	0.82 (0.75–0.88)	5.50 (4.70–6.30)	0.92 (0.88–0.95)	NS
2: Letter features	4.25 (4.05–4.45)	4.30 (4.10–4.50)	0.75 (0.68–0.82)	4.40 (4.20–4.60)	0.81 (0.74–0.87)	NS
3: Applicant abilities	2.58 (2.52–2.64)	2.58 (2.52–2.64)	0.70 (0.62–0.78)	2.60 (2.54–2.66)	0.77 (0.69–0.84)	NS

ICC, intraclass correlation coefficient; NS, nonsignificant.

The covariates of medical school, gender, and year of graduation were similar between the case and control groups, but small sample sizes in the medical school and year of graduation categories limited the ability to detect statistical differences (Table 3). Out of 15 residents in the case group, seven (46.7%) had deficiencies in medical knowledge and five (33.4%) in patient care. Additionally, four residents (26.7%) in the case group exhibited deficiencies in more than one competency.

**Table 3.** Study Participants' Baseline Characteristics

Characteristic	Case Group	Control Group	*P*
Gender			
Female	6 (40)	4 (26.7)	NS
Male	9 (60)	11 (73.3)	NS
Medical school			
A	2 (13.3)	2 (13.3)	NS*
B	1 (6.7)	1 (6.7)	NS*
C	2 (13.3)	2 (13.3)	NS*
D	3 (20)	1 (6.7)	NS*
E	1 (6.7)	2 (13.3)	NS*
F	2 (13.3)	3 (20)	NS*
H	1 (6.7)	1 (6.7)	NS*
I	1 (6.7)	3 (20)	NS*
W	2 (13.3)	1 (6.7)	NS*
Year of graduation			
2013–2015	5 (33.3)	5 (33.3)	NS*
2016–2018	5 (33.3)	4 (26.7)	NS*
2019–2021	4 (26.7)	5 (33.3)	NS*
2022–2023	1 (13.3)	1 (13.3)	NS*

NS, not significant.

Data are n (%).

* Due to the small sample size in these categories, there is insufficient power to generalize the nonsignificant conclusions.

In the first section of the rubric, there was a statistically significant difference between the mean score for participants in the case group (4.49; 95% CI, 3.29–5.68) and the control group (6.00; 95% CI, 5.00–7.00) (U=772.0, *P*=.017). Cohen's d was −0.41, indicating a medium effect size. When we performed a subgroup analysis of residents in the case group with more than one academic deficiency, the difference in mean scores was bigger (case group: 2.15; 95% CI, 1.42–3.38; control group: 6.00; 95% CI, 5.00–7.00) (U=120.5, *P*<.001). Cohen's d was −1.28, indicating a large effect size and suggesting a dependent effect between low letter scores and the presence of future academic deficiencies (Table 4).

**Table 4.** Analysis of Section 1 of the Rubric

Study Group	Score [Mean (95% CI)]	IQR	*P* (U Value)	Cohen's d
Control group (no deficiencies) (n=15)	6.00 (5.00–7.00)	5.60–6.80	.017	
Case group				
1 deficiency (n=15)	4.49 (3.29–5.68)	3.15–5.50	.02 (772.0)	−0.41
Multiple deficiencies (n=4)	2.15 (1.42–3.38)	1.88–2.30	<.001 (120.5)	−1.28

IQR, interquartile range.

For the second section of the rubric, there was no statistically significant difference between the mean overall score for participants in the case group (4.14; 95% CI, 3.91–4.38) and the control group (4.23; 95% CI, 4.05–4.40) (U=1,024.0, *P*=.933). The effect size was calculated as Cohen's d=−0.05, indicating a negligible effect and suggesting that letter features, alone, are not strong predictors of academic deficiencies (Appendix 5, available online at http://links.lww.com/AOG/D926).

There was no statistically significant difference between the mean overall score for participants in the case group (2.58; 95% CI, 2.55–2.61) and the control group (2.55; 95% CI, 2.52–2.58) (U=1,600.5, *P*=.160) for the last section of the rubric. However, for the individual ability “compassion”, there was a significant difference (case group: 2.74; 95% CI, 2.64–2.84; control group: 2.50; 95% CI, 2.38–2.62) (U=667.0, *P*=.005). The effect size was Cohen's d=0.24, indicating a small effect. No significant differences were found for any of the other abilities (Appendix 6, available online at http://links.lww.com/AOG/D926).

## DISCUSSION

When trained through a standardized process and integrated with a validated letter of recommendation scoring rubric, ChatGPT can provide reliable, efficient, and scalable analysis of letters of recommendation much faster than human reviewers. Our study also found a statistically significant difference in letter of recommendation scores between residents with and without academic deficiencies, indicating that AI can enhance the predictive value of letters of recommendation.

ChatGPT significantly reduced review time compared with human reviewers while producing scores that closely align with our benchmarks across all rubric sections. Initial prompts showed moderate to strong reliability, which improved with iteration and refinement. Our findings are consistent with Saudek et al's original validation study, which reported the highest reliability in section 1 of the rubric. However, ChatGPT achieved higher reliability across all sections, likely due to its consistent, unbiased application of scoring criteria. These results suggest that integrating AI tools such as ChatGPT with structured rubrics and continuous refinement can greatly enhance the reliability and efficiency of scoring letters of recommendation.

**Fig. 1.**
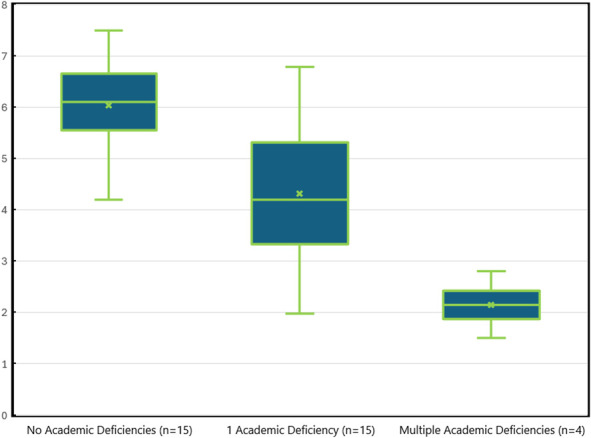
Box plots of mean scores for section 1 of the rubric are presented for individuals in the *control group* (residents without academic deficiencies) and individuals in the *case group* (residents with one or more than one deficiencies). The *box* represents the first and third quartiles (interquartile range [IQR] 5.60–6.80 for individuals in the control group, 3.15–5.50 for individuals in the case group with one deficiency, and 1.88–2.30 for individuals in the case group with multiple deficiencies), with the *band inside the box* representing the median scores (6.10 for individuals in the control group, 4.70 for individuals in the case group with one deficiency, and 2.15 for individuals in the case group with multiple deficiencies). *Whiskers* extend to the data within 1.5 times the IQR of the lower and upper quartiles or to the minimum and maximum values if no data exist beyond that range, specifically from 4.20 to 7.50 for individuals in the control group, 1.98 to 6.78 for individuals in the case group with one deficiency, 1.50 to 2.80 for individuals in the case group with multiple deficiencies. No significant outliers were observed in any group.

Despite mixed evidence on the predictive value of letters of recommendation,^22,25–30^ our study found a statistically significant difference in section 1 scores between residents with and without academic deficiencies, with a more pronounced effect in those with multiple deficiencies (Fig. 1). This suggests that ChatGPT-generated scores may offer greater predictive value for at-risk residents and offer the opportunity for early identification of individuals who may benefit from additional educational support. These findings contrast with those of Tamakuwala et al^27^ and Gudgel et al,^28^ who found letters of recommendation not predictive of resident performance, likely due to their lack of validated scoring instruments. Although Stohl et al^22^ found letters of recommendation predictive only at extremes, their study relied on hypothetical academic outcomes. In contrast, our study used comprehensive assessments by a Clinical Competency Committee, incorporating evaluations, peer and 360 feedback, case logs, and examination scores, providing a more robust measure of resident performance.

Our study supports the use of AI to reliably identify the “hidden code” in letters of recommendation reported by other authors. Research has shown that certain phrases are consistently interpreted among program directors. For example, Saudek et al^31^ found that phrases such as, “I give my highest recommendation” are rated positively, whereas, “showed improvement” is rated negatively. Rajesh et al^32^ also highlighted phrases such as, “We will plan to recruit this candidate” as strong endorsements. This may explain why differences in scores between the case and control groups were observed only in section 1 of the rubric, which examines phrases identified in prior research with program directors. Sections 2 and 3, related to letter features and applicant abilities, showed no differences between residents with and without academic deficiencies, aligning with research suggesting that letters of recommendation contain cues ChatGPT can consistently identify.

One of the major strengths of our study is its methodology, firmly grounded in prior research. We employed a published qualitative research framework to develop and refine ChatGPT prompts, ensuring a structured and systematic approach. The integration of a validated letter of recommendation scoring rubric added consistency, and continuous iteration and human oversight further enhanced the process. Our comprehensive definition of resident performance, based on Clinical Competency Committee assessments, offers a more reliable measure than the subjective metrics used in other studies. Additionally, our case–control design, with a powered sample size and control for potential confounders, enhances the validity of our results.

Several limitations should be acknowledged. First, conducting our study at a single institution may limit its generalizability. However, the validated rubric incorporated interpretations from program directors in various specialties, offering a solid framework adaptable to different programs. Although we matched participants in the control group for three potential confounders, unrecognized variables, such as the backgrounds of letter writers, still may have influenced the results. Finally, although ChatGPT required training for our specific analysis and some variability was observed across different accounts, the tool's ability to provide explanations about its decision-making process ensures transparency and allows for human review. It is important to note that, although ChatGPT offers valuable insights into the content of letters of recommendation, it is intended to complement, not replace, human reviewers. The tool provides objective data to assist program directors in making more informed decisions, but final judgments still will require human expertise and contextual interpretation (Fig. 1).

*Morales-Ramirez. ChatGPT for Analysis of Letters of Recommendation. O&G Open 2024*.

Future research should expand these findings by including residents from various specialties and institutions, exploring ChatGPT's effectiveness in other application sections, such as medical student performance evaluations and personal statements. Longitudinal studies tracking residents' performance over time could provide deeper insights into the predictive validity of letters of recommendation analyzed by ChatGPT. Further research should focus on developing standardized protocols for training and oversight of ChatGPT and comparing its performance with other LLMs. Although this study focused on ChatGPT's ability to replicate human-generated benchmark scores, future research should explore direct comparisons between ChatGPT and human reviewers in terms of predictive ability and inter-rater reliability.

In conclusion, ChatGPT can significantly enhance the resident selection process by enabling a more efficient and reliable analysis of letters of recommendation. Although our study indicates that ChatGPT still requires human training and oversight, it dramatically reduced the time required for reviewing letters of recommendation and provides results closely aligned with predefined benchmarks. By integrating ChatGPT into the selection process, programs can achieve a more efficient and objective review, ultimately improving the overall quality of candidate assessments.

## References

[1] Stone CL Dogbey GY Falls J Kuo YP. Key factors for residency interview selection from the National Resident Matching Program: analysis of residency Program Director surveys, 2016–2020. J Osteopathic Med 2023;123:523–30. doi: 10.1515/jom-2022-0144. 37615082

[2] Pershing S Co JPT Katznelson L. The new USMLE step 1 paradigm: an opportunity to cultivate diversity of excellence. Acad Med J Assoc Am Med Colleg 2020;95:1325–8. doi: 10.1097/ACM.0000000000003512 32433311

[3] Manstein SM Laikhter E Kazei DD Comer CD Shiah E Lin SJ. The upcoming pass/fail USMLE step 1 score reporting: an impact assessment from medical school deans. Plast Surg (Oakv) 2023;31:169–76. doi: 10.1177/22925503211034838 37188137 PMC10170630

[4] Johny A Shenot PJ Green C Chisholm L Riggs S Jackman SV Mp25-05 program directors' perspectives on the assessment of residency applicants in the post-USMLE step 1 era: a strong case for standardized letters of recommendation? J Urol 2023;209(suppl 4):e344. doi: 10.1097/JU.0000000000003253.05 38526424

[5] Trifoi M Claire Hollins L. The USMLE step 1 transitions to pass/fail scoring: perceptions of dermatology residents. J Skin 2023;7:631–4. doi: 10.25251/skin.7.1.14

[6] Dirschl DR Adams GL. Reliability in evaluating letters of recommendation. Acad Med 2000;75:1029. doi: 10.1097/00001888-200010000-00022 11031153

[7] Girzadas DV Jr Harwood RC Dearie J Garrett S. A comparison of standardized and narrative letters of recommendation. Acad Emerg Med 1998;5:1101–4. doi: 10.1111/j.1553-2712.1998.tb02670.x 9835474

[8] Kaffenberger J Mosser JL Lee GL Pootrakul L Harfmann K Fabbro S A retrospective analysis comparing the new standardized letter of recommendation in dermatology with the classic narrative letter of recommendation. J Clin Aesthet Dermatol 2016;9:36–42. PMC5110327 27878060

[9] Kang HP Robertson DM Levine WN Lieberman JR. Evaluating the standardized letter of recommendation form in applicants to orthopaedic surgery residency. J Am Acad Orthop Surg 2020;28:814–22. doi: 10.5435/JAAOS-D-19-00423 31868837

[10] Feldman MJ Ortiz AV Roth SG Dambrino RJ Yengo-Kahn AM Chitale RV An examination of standardized letters of recommendation rating scales among neurosurgical residency candidates during the 2020-2021 application cycle. Neurosurgery 2021;89:1005–11. doi: 10.1093/neuros/nyab346 34624075 PMC8600167

[11] Zhang H Wu C Xie J Lyu Y Cai J Carroll JM. Redefining qualitative analysis in the AI era: utilizing ChatGPT for efficient thematic analysis. Preprint. 2023. doi: 10.48550/ARXIV.2309.10771

[12] Bano M Zowghi D Whittle J. Exploring qualitative research using LLMs. Preprint. Posted online June 23, 2023. doi: 10.48550/arXiv.2306.13298

[13] Vasan V Cheng CP Lerner DK Pascual K Mercado A Iloreta AM Machine learning for predictive analysis of otolaryngology residency letters of recommendation. Laryngoscope 2024;134:4016–22. doi: 10.1002/lary.31439 38602257

[14] Drum B Shi J Peterson B Lamb S Hurdle JF Gradick C. Using natural language processing and machine learning to identify internal medicine–pediatrics residency values in applications. Acad Med J Assoc Am Med Colleg 2023;98:1278–82. doi: 10.1097/ACM.0000000000005352 37506388

[15] Mahtani AU Reinstein I Marin M Burk-Rafel J. A new tool for holistic residency application review: using natural language processing of applicant experiences to predict interview invitation. Acad Med J Assoc Am Med Colleg 2023;98:1018–21. doi: 10.1097/ACM.0000000000005210 36940395

[16] Vasan V Cheng C Lerner DK Signore AD Schaberg M Govindaraj S Letters of recommendations and personal statements for rhinology fellowship: a deep learning linguistic analysis. Int Forum Allergy Rhinol 2023;13:1971–3. doi: 10.1002/alr.23153 36896816

[17] John AS Kavic SM. Leveraging artificial intelligence for resident recruitment: can the dream of holistic review be realized? Art Int Surg 2022;2:195–206. doi: 10.20517/ais.2022.24

[18] Abbott KL George BC Sandhu G Harbaugh CM Gauger PG Ötleş E Natural language processing to estimate clinical competency committee ratings. J Surg Educ 2021;78:2046–51. doi: 10.1016/j.jsurg.2021.06.013 34266789

[19] Solano QP Hayward L Chopra Z Quanstrom K Kendrick D Abbott KL Natural language processing and assessment of resident feedback quality. J Surg Educ 2021;78:e72–7. doi: 10.1016/j.jsurg.2021.05.012 34167908

[20] Saudek K Treat R Rogers A Hahn D Lauck S Saudek D A novel faculty development tool for writing a letter of recommendation. PLoS One 2020;15:e0244016. doi: 10.1371/journal.pone.0244016 33326489 PMC7743943

[21] Blechman A Gussman D. Letters of recommendation: an analysis for evidence of Accreditation Council for Graduate Medical Education core competencies. J Reprod Med 2008;53:793–7. 19004407

[22] Stohl HE Hueppchen NA Bienstock JL. The utility of letters of recommendation in predicting resident success: can the ACGME competencies help? J Graduate Med Educ 2011;3:387–90. doi: 10.4300/JGME-D-11-00010.1 PMC3179231 22942969

[23] Drum B Lamb S Gradick C. Values-based resident selection in an internal medicine-pediatrics residency program. J Gen Intern Med 2023;38:1410–6. doi: 10.1007/s11606-022-07857-y 36344647 PMC10160323

[24] Koo TK Li MY. A guideline of selecting and reporting intraclass correlation coefficients for reliability research. J Chiropractic Med 2016;15:155–63. doi: 10.1016/j.jcm.2016.02.012 PMC4913118 27330520

[25] Schaverien MV. Selection for surgical training: an evidence-based review. J Surg Educ 2016;73:721–9. doi: 10.1016/j.jsurg.2016.02.007 27133583

[26] Zuckerman SL Kelly PD Dewan MC Morone PJ Yengo-Kahn AM Magarik JA Predicting resident performance from preresidency factors: a systematic review and applicability to neurosurgical training. World Neurosurg 2018;110:475–84.e10. doi: 10.1016/j.wneu.2017.11.078 29174240

[27] Tamakuwala S Dean J Kramer KJ Shafi A Ottum S George J Potential impact of pass/fail scores on USMLE step 1: predictors of excellence in obstetrics and gynecology residency training. J Med Educ Curricular Develop 2021;8:23821205211037444. doi: 10.1177/23821205211037444 PMC8597065 34805529

[28] Gudgel BM Melson AT Dvorak J Ding K Siatkowski RM. Correlation of ophthalmology residency application Characteristics with subsequent performance in residency. J Acad Ophthalmol 2021;13:e151–7. doi: 10.1055/s-0041-1733932 PMC9928014 37388830

[29] Brenner AM Mathai S Jain S Mohl PC. Can we predict “problem residents”. Acad Med J Assoc Am Med Colleg 2010;85:1147–51. doi: 10.1097/ACM.0b013e3181e1a85d 20592510

[30] Hartman ND Lefebvre CW Manthey DE. A narrative review of the evidence supporting factors used by residency program directors to select applicants for interviews. J Graduate Med Educ 2019;11:268–73. doi: 10.4300/JGME-D-18-00979.3 PMC6570461 31210855

[31] Saudek K Treat R Goldblatt M Saudek D Toth H Weisgerber M. Pediatric, surgery, and internal medicine program director interpretations of letters of recommendation. Acad Med J Assoc Am Med Colleg 2019;94:S64–8. doi: 10.1097/ACM.0000000000002919 31365410

[32] Rajesh A Rivera M Asaad M Chandra A Baloul MS Backstrom CM What are we really looking for in a letter of recommendation? J Surg Educ 2019;76:e118–24. doi: 10.1016/j.jsurg.2019.06.008 31302033

